# Alternative use of endocavitary probe to guide minimally invasive partial nephrectomy: is it reasonable?

**DOI:** 10.1590/acb370607

**Published:** 2022-09-19

**Authors:** Lucas Teixeira Batista, José Guilherme Reis de Oliveira, Vitor Parente Gouvea, Leonardo Azevedo de Souza, Rafael Tourinho-Barbosa

**Affiliations:** 1 PhD. Universidade Federal da Bahia – Department of Urology – Salvador (BA), Brazil.; 2 PhD. Hospital Cardiopulmonar – Department of Urology – Salvador (BA), Brazil.; 3 Graduate student. Escola Bahiana de Medicina e Saúde Pública – Salvador (BA), Brazil.; 4 PhD. Faculdade de Medicina do ABC – Department of Urology – Santo André (SP), Brazil.

**Keywords:** Nephrectomy, Kidney Neoplasms, Surgical Procedures, Operative, Ultrasonography

## Abstract

**Purpose:**

To describe the use of endocavitary ultrasound probe as an auxiliary tool when performing partial nephrectomy in cases of endophytic renal tumors, to standardize the method, and to report the preliminary results achieved with this technique.

**Methods:**

Fifteen patients diagnosed with completely endophytic underwent partial nephrectomy with the use of an endocavitary ultrasound probe. This article describes the technique involved in partial nephrectomy and details the preparation of the endocavitary ultrasound probe to ensure its safe use.

**Results:**

All the patients had a RENAL score between 8 and 11. The median time of warm ischemia was 26 and 18 minutes for laparoscopic or robot-assisted surgery, respectively. The median duration of surgery was 150 minutes, and the median console time was 145 minutes for the laparoscopic and robot-assisted surgery groups, respectively. The median estimate of blood loss was 200 mL. Only three patients in the laparoscopic group had focal positive surgical margins. There were no cases of infection at the site of probe entry.

**Conclusions:**

Intraoperative use of an endocavitary ultrasound probe for partial nephrectomy is possible and a safe alternative to the excision of endophytic tumors when neither robotic probes nor laparoscopic probes are available.

## Introduction

Partial nephrectomy should always be an option to treat T1 renal tumors (7 cm or less)^1^. Nephron sparing surgery is the best alternative to preserve overall renal function and reduce cardiovascular morbidity, with a potential effect on overall survival^2^. Minimally invasive nephrectomy is the procedure of choice for the treatment of T1 tumors. Advantages of this procedure include lower postoperative morbidity, no negative impact on oncologic outcome and faster return to work after surgery^3^^-^ 5.

With the advent of robotic surgery and a simultaneous improvement in imaging methods, partial nephrectomy, initially offered for less complex tumors, began to be used for increasingly complex lesions^6^. Intraoperative ultrasonography constitutes an important auxiliary tool in the treatment of endophytic renal masses since it allows the demarcation of the tumor position and definition of the relationship between the tumor and the vessels of the renal hilum and the pyelocaliceal system. These advantageous features enable complex renal tumors to be resected^7^.

The use of laparoscopic or robot-assisted ultrasonography is recommended; nevertheless, the high cost involved constitutes a drawback^8^^,^ 9. Conversely, an endocavitary ultrasound probe represents a cheap and accessible alternative. Nonetheless, although widely available and extensively used, there are few papers in the literature dealing with the standardization of the technique or the training and learning curve associated with the use of endocavitary probes in partial nephrectomy.

The objectives of the present study were to describe the technique involved in the use of an endocavitary ultrasound probe as an auxiliary tool when performing partial nephrectomy in cases of endophytic renal tumors and to report the preliminary results achieved with the use of this technique.

## Methods

This study was conducted with 15 patients diagnosed with a renal tumor < 7 cm, which was completely endophytic. The patients underwent minimally invasive partial nephrectomy (laparoscopic or robot-assisted) with the use of an endocavitary ultrasound probe between 2017 and 2019. The techniques involved in minimally invasive partial nephrectomy and in the safe and efficient use of endocavitary ultrasound probes are described here in detail.

Data collected preoperatively included body mass index (BMI), renal function and RENAL nephrometry score. Intraoperative data included the duration of surgery, intraoperative bleeding, warm ischemia time, conversion rate, intraoperative complications, and opening of the pyelocaliceal system. All the patients were followed up prospectively, at least for two years, with data on positive surgical margins, perioperative complication rates, and renal function being recorded.

The ultrasound used in this study was the Acuson X300 Premium Edition (Siemens Medical Solutions USA, Inc.), with a Acuson EC9-4 convex endocavitary ultrasound probe (Siemens Medical Solutions USA, Inc.) with a 38-mm-width array and frequency range of 4-9 MHz.

Continuous variables were reported as medians and interquartile ranges (IQR) and categorical variables as absolute numbers and percentages. Although the data refer to the total number of patients undergoing laparoscopic or robot-assisted partial nephrectomy, no comparative analysis was conducted between these two groups due to the lack of statistical power of this sample.

Verbal or written consent could not be obtained because the study was a file scanning study, and retrospective and no changes were made in the management.

### Surgical technique

The patients were placed in the lateral decubitus position at 30-degrees following general anesthesia. Pneumoperitoneum was achieved using a Veress needle, and transperitoneal access was attained. Surgery was performed using laparoscopic or robot-assisted techniques, as available. In the patients submitted to laparoscopy whose tumor was on the left kidney, one 12-mm trocar, one 10-mm trocar and two 5-mm trocars were used, whereas in the robot-assisted laparoscopies two 12-mm and three 8-mm robotic trocars were used. In the case of tumors on the right kidney, an additional 5-mm trocar was used to retract the liver in both techniques.

After exposure of Gerota’s fascia, a direct approach to the renal pedicle was performed before opening the fascia. In hilar tumors, the renal artery and vein were isolated, and their branches and tributaries were dissected as far as the renal sinus. After dissection of the renal hilum, Gerota’s fascia was opened and the kidney was completely freed, irrespective of the tumor site. This allows better renal mobility, enabling the endocavitary ultrasound probe to be correctly positioned.

### Preparation of the endocavitary probe

**Figure 1:**
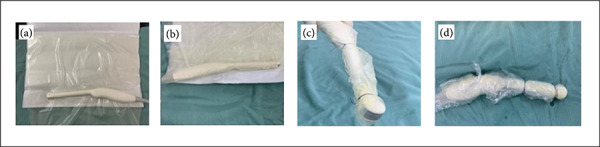
Preparation of the endocavitary probe.

A clean, non-sterile probe was placed in the surgical field on an open Steri-Drape plastic film (3M, St. Paul, MN, United States of America), and xylocaine jelly was placed on its tip (Fig. 1a). Next, the Steri-Drape was closed, completely enclosing the probe (Fig. 1b), with any excess film being removed to minimize the increase in the probe diameter. Subsequently, the Steri-Drape was secured at its distal and proximal ends using cotton suture thread (Fig. 1c). The package was then enclosed in a sterile latex sheath, thus creating a double layer of protection. This outer sheath was also secured at both ends using cotton suture thread (Fig. 1d).

### Access and positioning of the probe

**Figure 2:**
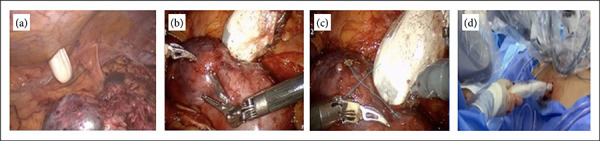
Access and positioning of the endocavitary probe.

After Gerota’s fascia was opened and the kidney totally dissected, the probe was inserted through a posterior counterincision below the 12^th^ rib, directing at a right angle to the tumor site. The incision should be between 2 and 3 cm in length to prevent loss of pneumoperitoneum. The muscle planes were dissected up to the parietal peritoneum, guided by laparoscopic vision. Next, the tunnel was dilated by the surgeon’s index finger (Fig. 2a), and the probe was carefully inserted (Fig. 2b). Incision of the skin and dissection of the muscle planes should be performed at a favorable angle to enable the probe to be adequately positioned over the tumor.

**Figure 3:**
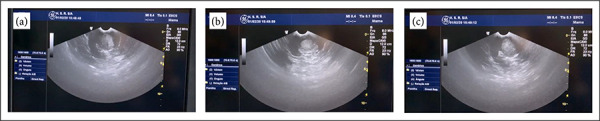
Endocavitary probe image projected onto the ultrasoundcart screen with visualization of the renal endophytic tumor.

The probe was placed over the kidney, and the tumor was identified using Doppler velocimetry. The margins and depth of the tumor, as well as its distance from the capsule and from the renal sinus, were measured. To correctly identify the tumor margin, the endocavitary probe should be positioned over the lesion until the normal renal parenchyma was identified and then demarcated using a monopolar scalpel (Fig. 2c). A radiologist was responsible to manipulate the ultrasound (Fig. 2d), and the image was projected onto the ultrasound cart screen (Figs. 3a-3c). The tumor was then outlined circumferentially, and the distances to the capsule and renal sinus were recorded.

After the demarcation of the tumor, the probe should be withdrawn, but not completely removed from the cavity, so as to block the entry incision and allow further evaluation of the tumor site in case of doubts during and after excision of the tumor. If venous tumoral thrombosis is suspected, the probe can be used as a diagnostic tool over the renal vein and vena cava. Partial nephrectomy should only proceed if the diagnostic investigation is negative. During the procedure, the surgeon and the assistant surgeon should be careful to avoid any inadvertent damage to the endocavitary ultrasound probe.

### Excision of the tumor and renorrhaphy

The principal renal artery was clamped, with selective clamping being reserved for selected cases. The renal vein is only clamped when the tumor is situated in the renal hilum, close to the principal renal vein or tributaries. In complex tumors on the right kidney, renal-vein clamping is also performed to reduce reflux bleeding, permitting better visualization of the resection plane. The previously demarcated renal margin was resected using cold scissors, and the tumor was enucleated to avoid damaging adjacent structures. The vessels that nourish the tumor were ligated using polymer clips and resected. The integrity of the tumor pseudocapsule and the surgical margin were continuously evaluated macroscopically during the excision of the tumor.

Renorrhaphy was performed on two or more planes using poliglecaprone or polydioxanone 2.0 suture with an atraumatic needle. Continuous suturing was performed on the renal medulla, closing the pyelocaliceal system and vessels in the renal sinus. A new line of the suture is performed on the renal medulla if the area of the tumor bed is large or if the vessels of the pyelocaliceal system remain open. A polymer clip was placed at each end of the thread, at its entrance and exit through the renal cortex. Traction should be performed on the suture lines in the renal medulla, which should be secured using another polymer clip, thus ensuring that the edges of the area resected were pulled together. Next, the cortex was sutured using separate sutures in an X shape with the same type of suture thread used for the renal medulla. Polymer clips were placed at both ends of the thread. The vessels of the hilum were unclamped at an early stage before the cortex was sutured. Since bleeding is usually slight, hemostatic agents are rarely used.

## Results

Fifteen patients with a renal tumor with endophytic growth pattern underwent minimally invasive partial nephrectomy guided by an endocavitary ultrasound probe. The same surgeon, experienced in performing minimally invasive surgery, carried out all the surgical procedures, seven by laparoscopy and eight in the form of robot-assisted surgery. The median age of the patients was 68 years old (interquartile range [IQR] = 62-72), and median BMI was 27 Kg/m2 (IQR = 25-29). All the tumors were completely endophytic, with a median size of 30 mm (IQR = 25-33 mm). In relation to the complexity of the lesion, seven patients had a RENAL score of 8-9 (moderate complexity), and eight patients had a score of 10-11 (high complexity). Seven patients had anterior renal lesions, seven had posterior lesions, and one had a lateral lesion. Table 1 shows the characteristics of the patients and the renal tumors at baseline according to the surgical technique used.

**Table 1:** Descriptive characteristics of 15 patients with endophytic tumors who underwentpartial nephrectomy guided by endocavitary ultrasound probe.

Patient description	Overall sample population(n = 15)	Laparoscopy group(n = 7)	Robotic group (n = 8)
	Median (interquartile range)		
Age (years)	68 (62-72)	65 (58-68)	64 (47-74)
Body mass index (kg/m^2^)	27 (25-29)	27 (24-29)	27 (26-29)
Tumor size (mm)	30 (25-33)	30 (27-32)	29 (19-37)
***Tumor location***	**N (%)**		
Anterior	7 (47)	3 (43)	4 (50)
Posterior	7 (47)	4 (57)	3 (38)
Lateral	1 (6.7)	0	1 (12)
***Tumor complexity (RENAL nephrometry score)***	**N (%)**		
8	4 (27)	1 (14)	3 (38)
9	3 (20)	1 (14)	1 (12)
10	4 (27)	2 (29)	1 (12)
11	4 (27)	3 (43)	3 (38)

The median time of warm ischemia was 26 minutes (IQR = 18-29 minutes) and 18 minutes (IQR = 16-25 minutes) for the patients submitted to laparoscopic surgery or robot-assisted surgery, respectively. The median duration of surgery was 150 minutes (IQR = 150-160 minutes), and the median console time was 145 minutes (IQR = 120-165 minutes) for the laparoscopic and robot-assisted surgery groups, respectively. Median estimate blood loss was 200 mL (IQR = 150-300 mL) for the entire group of patients, and 200 mL (IQR = 150-300 mL) and 175 mL (IQR = 150-300 mL) for the laparoscopic and robot-assisted surgery groups, respectively.. Only one patient, submitted to laparoscopy for an angiomyolipoma, with a RENAL score of 10, had more extensive bleeding (500 mL); however, blood transfusion was not required. In that patient, creatinine levels increased by 20% compared to preoperative levels, but returned to baseline levels after six months. Only two patients in the laparoscopic group had focal positive surgical margins. No other patient had any perioperative complication, and there were no other cases of bleeding > 300 mL, no need for blood transfusion, and no patient had an increase in creatinine levels > 20%. None of the patients required surgical conversion. There were no cases of infection at the site of probe entry.

## Discussion

Oncologic outcome and recovery of renal function following minimally invasive partial nephrectomy are similar to those achieved with open surgery^10^^,^ 11. Advantages of the procedure include reduction in hospital stay and in perioperative complication rates.

Treating endophytic renal tumors is challenging, since their characteristic intrarenal location does not allow anatomic limitations to be defined. The use of ultrasonography during surgery is recommended in order to adequately plan for a procedure in which warm ischemia makes excision of the tumor time-dependent^12^. Studies suggest that partial nephrectomy for endophytic tumors using intraoperative ultrasonography achieves oncologic results and complication rates similar to those found in patients with exophytic lesions; however, the duration of surgery is longer due to the difficulty in resecting the tumor^13^^-^ 15.

Ultrasonography with a robotic probe offers additional benefits when compared to an endocavitary ultrasound probe^16^. The ultrasound image from the robotic probe is projected onto the console screen; therefore, the surgeon does not have to move away to see the image on an external screen. Furthermore, the robotic probe is inserted using a 5 or 12 mm trocar, with no need for a further incision of greater diameter. The robotic probe is flexible, linear, and adjustable to the robot forceps, allowing a greater range of movement, better control for the surgeon and better angles at which to visualize the tumor. In medical centers where there is a robotic platform but no robotic probe, laparoscopic probes have been used with similar results^17^. In a significant proportion of hospitals in developing countries, however, these devices are unavailable due to their high cost. Therefore, the use of an endocavitary ultrasound probe is proposed as a cheaper alternative. Nonetheless, few studies have been published in the literature on the standardization of techniques regarding the access and manipulation of endocavitary ultrasound probes during partial nephrectomy.

This paper describes the standardization of the technique of partial nephrectomy guided by an endocavitary ultrasound probe for cases of endophytic tumors. The principal aspects associated with the technique are: direct approach of the renal hilum, dissection of branches and tributaries of the renal vessels in the case of renal hilar tumors, complete release of the kidney from Gerota’s fascia to permit mobilization in the direction of the endocavitary probe, demarcation of the tumor edges using a monopolar scalpel, measurement of the distance from the tumor to the capsule and renal sinus, dissection of as much of the tumor as possible before clamping the hilum to reduce the time of warm ischemia, clamping the renal vein in cases of hilar tumors or complex tumors on the right kidney, and tumor enucleation, thus avoiding damage to adjacent structures.

There are some limitations associated with the use of the endocavitary ultrasound probe in relation to robotic probes and laparoscopic probes. The contact surface of the endocavitary ultrasound probe is convex, hampering appropriate attachment to the surface of the kidney, and the visualization window is smaller. Furthermore, the acquisition of images depends on the radiologist. To guarantee a satisfactory ultrasound image, complete mobilization of the kidney is essential. In addition, the probe must be correctly positioned at a favorable angle to the position of the tumor, in which the tumor should be located opposite the probe. Since performing ultrasonography and simultaneously carrying out excision is impossible with an endocavitary ultrasound probe, it is essential that the tumor is demarcated and the distance from the tumor to the capsule and renal sinus measured.

The endocavitary ultrasound probe must be adequately prepared to prevent infection at the insertion site, while guaranteeing the quality of the ultrasound image. The probe should be covered with a double sterile layer and inserted delicately through a wide incision to avoid tearing the casing. In the 15 surgeries reported here, there was no tearing of the protective layers of the probe or infection at the insertion site.

## Conclusions

Intraoperative use of an endocavitary ultrasound probe for laparoscopic or robot-assisted partial nephrectomy is possible and simple to learn as long as a standardized technique is established. The use of this technique has proven to represent a safe alternative for the excision of endophytic tumors in medical centers where neither robotic probes nor laparoscopic probes are available.
